# Supports for and Barriers to Healthy Living for Native Hawaiian Young Adults Enrolled in Community Colleges

**Published:** 2007-09-15

**Authors:** Jamie K Boyd, Kathryn L Braun

**Affiliations:** University of Hawaii, Windward Community College; University of Hawaii, Public Health, and ‘Imi Hale – Native Hawaiian Cancer Awareness, Research and Training Network, Honolulu, Hawaii

## Abstract

**Introduction:**

Physical inactivity and lower levels of education are associated with increased risk for obesity and chronic disease. Compared with other racial/ethnic groups in Hawai'i, Native Hawaiians have a higher prevalence of chronic disease, including diabetes, cancer, and cardiovascular disease. In 2000, 72.5% of Native Hawaiians were overweight, 54.4% met national recommendations for physical activity, and about 10% enrolled in college.

**Methods:**

We conducted four focus groups involving 32 Native Hawaiian young adults enrolled in community (i.e., 2-year) colleges to explore perceived supports for and barriers to living a healthy lifestyle. Questions were based on social marketing concepts and proven physical activity strategies. We adhered to cultural protocol and engaged 10 key informants to help develop the study. Results of the study were presented to these key informants.

**Results:**

Native Hawaiian young adults perceive themselves as invincible and cited demanding lifestyle and laziness as barriers to increasing their levels of physical activity. Young adults did not define health in terms of individual strength, endurance, and appearance. Rather, they defined it in terms of being purposefully engaged in life's responsibilities, which include working, going to school, and caring for family. Native Hawaiian young adults expressed preferences for group-oriented and college-course–based opportunities to learn more about healthy living and to be encouraged to become more physically active.

**Conclusion:**

Our research provides insights into the barriers to and supports for increasing physical activity levels among Native Hawaiian young adults and confirms the importance of talking to targeted end-users before designing interventions.

## Introduction

Pacific Islanders (including Native Hawaiians) generally experience poorer health than the rest of the U.S. population (1). In Hawai'i in 2005, Native Hawaiians comprised about 22% of the population; other major racial groups represented were white (24%), Japanese (24%), and Filipino (16%) (2). Compared with these other groups, Native Hawaiians experience disproportionately high rates of mortality from many chronic diseases (3,4), including cancer (5-7). In 2000, only 54.4% of Native Hawaiians living in Hawai'i met national recommendations for physical activity, and 72.5% were overweight (body mass index ≥25.0 kg/m^2^) (5,8).

In addition to having poor health, Native Hawaiians experience low rates of high school graduation and college enrollment (2,3). Although 22% of Hawai'i's population is Native Hawaiian, less than 10% of students enrolled in Hawai'i colleges are of Hawaiian ancestry (9). Residents of Native Hawaiian ancestry are more likely to hold lower-paying jobs than residents of other races/ethnicities in Hawai'i (2,3), probably because of their lower high school graduation and college enrollment rates.

Data on college students (i.e., young adults aged 18–25 years) gathered through the Youth Risk Behavior Surveillance System (YRBSS) suggest that minority students and students in community (i.e., 2-year) colleges are less likely to receive important health information than are white students and students in 4-year colleges (10). This disparity in health education is concerning given the known associations between minority status and lower educational level, lower socioeconomic status, chronic conditions, and shorter life expectancy (11-14). Because of these associations, research to improve quality of life and life expectancy indicates that it is especially important to focus primary prevention efforts on people at risk for low educational attainment (14).

Reports of successful primary prevention strategies developed and tested with minority populations across the lifespan are abundant (15-18). However, few studies have assessed the health perceptions and supports and barriers important in planning prevention programs to help college students to live a healthy lifestyle during the college years and later (19,20). Research also confirms the importance of considering cultural preferences and practices when adapting programs to a new target group (21-23). Studies are needed to assess ways to adapt evidence-based strategies to new populations (24), including young adult college students.

Many good reasons exist to explore primary prevention strategies for young adults attending community colleges. Individuals in this group are in transition between adolescence and adulthood and are beginning to make independent decisions that affect long-term health and educational outcomes (19). They are likely to be minority students, have fewer socioeconomic resources than students in 4-year colleges, and have work and family responsibilities. Given that young adults are expressing motivation to make a positive life change by enrolling in college, community colleges are important venues for health promotion programs. The purpose of this study was to learn about the perceptions of healthy lifestyles, supports for and barriers to healthy living, and recommendations for desirable program planning among Native Hawaiian young adults enrolled in community colleges.

## Methods

### Design

This study was part of a multisite study undertaken by Community Networks Program grantees of the National Cancer Institute's Center to Reduce Cancer Health Disparities (25-26). We studied Native Hawaiian community college students while other researchers studied Hmongs in California, African Americans in the Mississippi Delta, and Hispanics in Texas. All four sites explored the "four P's" of a social marketing framework to guide focus groups: 1) product — what the consumer is asked to "buy" (in this case, healthy behaviors); 2) price — the monetary, psychological, emotional, and time costs; 3) place — how and where the consumer uses the product; and 4) promotion — how information about the product reaches the target audience and how promotion activities attract the intended audience (27-29). Each site also involved key informants (i.e., gatekeepers) (25).

The Hawai'i study was conducted at Leeward Community College (LCC), one of seven community colleges in the 10-campus University of Hawai'i system. Of the seven 2-year colleges, four are on the island of O'ahu where 72% of the state's population lives (30). LCC is located in rural west O'ahu, home to the highest concentration of Native Hawaiian residents on the island (31). The study was approved by the University of Hawai'i and the Native Hawaiian Health Care System Institutional Review Boards.

### Participants

Focus group inclusion criteria included self-reported Native Hawaiian ancestry (i.e., ability to trace ancestry to before 1778 when Captain James Cook claimed the islands for England), being a current resident of O'ahu, Hawai'i, being between 18 and 25 years of age, being currently enrolled in a community college, and not being concurrently enrolled in a health promotion program. Thirty-two students participated.

Key informants were volunteer members of a community action group (CAG) assembled for this study. Selected community members had experience in health promotion, college activities, and/or Native Hawaiian culture. We included *kūpuna* (i.e., elders), neighbors, and professionals, as recommended by the 1998 Native Hawaiian Health and Wellness Summit and Island *'Aha* report (32). Specifically, the report advocated for *ola hāloa* (i.e., sustaining of life) through expression of the cultural value of *ho'oLōkahi* (i.e., bringing about unity) by building "collaboration and alliance between researchers and the community" (29). The use of key informants also helped ground the study in the community (33). All 10 key informants were long-term Hawai'i residents, seven were Native Hawaiian, four were health professionals (e.g., physician, nurse, social worker), four were program or college administrators, and two were college students. Four key informants were men and six were women. They ranged in age from 25 to 72 years, and four were considered to be *kūpuna*.

### Procedures

To recruit participants, research associates visited courses with high Native Hawaiian enrollment (e.g., Hawaiian Studies classes) at the four community colleges on O'ahu to describe the purpose of the research and participant recruitment criteria. Interested students were invited to telephone the project office. During the telephone call, details of the study and inclusion criteria were reviewed, and eligible students were scheduled to attend a focus group. The goal was to recruit 14 participants per session to achieve an attendance count of 6 to 10 students. Two reminder phone calls were made; the first occurred one week before the study and the second on the day before the group meeting. Written consent to participate was obtained before each focus group.

Research staff was trained in focus group procedures by the coinvestigator (K.L.B.) from 'Imi Hale – Native Hawaiian Cancer Awareness, Research and Training Network (21), following a guide developed by the CAG. Trainees included the principal investigator (PI), coordinator, moderator, transcriptionist, and research assistant. Focus groups were conducted in 2005 at LCC in a private recording studio and were video and audio recorded by trained faculty. All focus group sessions were led by the trained moderator while the PI (J.K.B.) watched from the video control room. Student discomfort with the auditorium-style seating used in the first focus group led us to rearrange seating to a closed circle, and we also gave students more pretaping time in the studio to increase their comfort with one another and the setting. Five sessions were scheduled, but only four (with a total of 32 participants) were necessary to obtain the volume of data sufficient to reach saturation. The PI and moderator debriefed each other immediately after each session, noting insights about group agreement or disagreement (including head nodding and head shaking), laughter, emotional interactions, and silence. These notes were incorporated into the transcripts.

### Measures

A brief, researcher-designed questionnaire was used to gather demographic data. Participants rated their health on a 6-point scale ranging from "very unhealthy" to "very healthy" and provided information about how much exercise they engaged in each week. Participants completed the questionnaire before participating in the focus group sessions. The CAG used the audience-based marketing framework to design a focus group guide that would elicit information from the target audience about attitudes regarding healthy lifestyles and about audience members' insights into products (primary prevention programs) that would be attractive, reasonably priced, and conveniently accessible for college students (27-29). Questions included

- What does being healthy mean to you? What makes it easy or hard to be healthy?
- When you hear the phrase "physical activity" or "being physically active," what does that mean to you?
- What kinds of [physical] activities do you do? What is it about these activities that make them more appealing to you than other activities? What makes it difficult for you to do other activities?
- What kinds of services, programs, or environmental supports [to encourage healthy living among young adults] would you recommend?

Following the last question, we specifically solicited comments on five core prevention strategies to increase physical activity identified by the Centers for Disease Control and Prevention (CDC)Following the last question, we specifically solicited comments on five core prevention strategies to increase physical activity identified by the Centers for Disease Control and Prevention (CDC). Core strategies included 1) implementing community-wide campaigns, 2) conducting individual behavior change programs, 3) creating or enhancing access to places for physical activity, 4) implementing social supports in community settings, and 5) using point-of-decision prompts (25,34). We also asked about the appeal of a college course to teach healthy lifestyle skills, if it had not already been mentioned by a focus group participant.

### Analysis

Video and audio recordings and program notes were used to produce unabridged transcripts for analysis. Transcript analysis was inductive. The PI, coinvestigator, and research assistant read the transcripts individually and as a team, one focus group at a time, adhering to a cyclic process of abstracting, comparing, and agreeing on themes. The cyclic process continued until no new themes emerged (i.e., a point of data saturation was reached). After researchers reached consensus on a framework that reflected a collective representation of themes, a codebook was developed. The three researchers then reread and coded the transcripts using the codebook. Codes and the individuals they were attributed to were compared, and disagreements were discussed until consensus was reached. Instances of potential bias were explored, and biased coding was excluded.

We then counted the number of individuals and groups that spoke to each theme. A long-table template was developed using a large (3 ft x 3 ft) computer-generated grid. The template was designed to enhance visual assessment of how many participants discussed the same theme. Specificity was determined not by the extensiveness of participants' responses but by the emphasis and detail of a specific response to a specific question. The visual tool enhanced grouping of common ideas (35).

Preliminary findings were summarized and presented to key informants in an open discussion format. This procedure is consistent with cultural protocol in which younger adults look to *kūpuna* to voice their *mana'o* (i.e., ideas and opinions) about how ideas and cultural concepts are interpreted (36,37). The PI made handwritten notes of each key informant's response to share with the coinvestigator. Key informant responses were then summarized by theme.

## Results

Three major themes emerged from the data analysis — ideal health, supports for and barriers to healthy living, and ideas for program planning — each with several subthemes. We first present the findings by theme for the students. We then provide the findings by theme for the key informants.

### Students

The mean student age was 20 years (data not shown), and most students were female, single, and working at least part time (Table 1). At the time of this study, most students were living with their families and resided on the rural Wai'anae Coast of O'ahu. These characteristics are comparable with those of the general LCC population, except that a higher proportion (19%) of focus group participants was enrolled in vocational career fields than was the proportion (6%) from the general LCC population, indicating a potential risk for low-socioeconomic status of these Native Hawaiians even after graduating from LCC. Self-rated health and exercise behaviors of student participants are shown in Table 2. Of the participants, three rated themselves as "somewhat unhealthy," six indicated they were "unsure," and most (21 of 32) rated themselves as "somewhat healthy." However, 19 of 32 students were not meeting the minimum recommendation for physical activity (i.e., at least 30 minutes of moderate-intensity physical activity on 5 or more days per week), including four who did not get any exercise (38).

#### Theme 1: Ideal health

Themes and subthemes generated during the focus groups are presented in Table 3. Three subthemes predominated under the ideal health theme: 1) being active through purposeful living (e.g., performing domestic chores, taking the stairs, conducting work-related activities), 2) having a good self-identity, and 3) following a healthy diet. Being active was seen as a key component of ideal health. However, few students defined health and physical fitness in terms of planned exercise (e.g., jogging, biking, working out at a gym), and only two participants spoke positively about the benefits of regular strenuous exercise. Rather, 20 of 32 students defined being active as “just moving around” while performing purposeful responsibilities of daily living. These responsibilities included domestic duties, as noted by several young female participants: “Like, I’ll be watching the kids, or I’ll take them out; I’m constantly moving around every day,” and “I spend most of my time with them [niece and nephew] and that’s a major source of exercise . . . going to the park or something.” Other students noted that they were physically active because they “walked to the store” or “walked to work.” Another said, “I think just as long as you do something during the day . . . I don’t know, just anything.”

Several of the students described being physically active through paid jobs. For example, a young man said, "I rely on my work . . . I'm up and down ladders constantly. It's not really a workout, but it is physical labor that keeps the blood flowing and breathing. For me, that is healthy."

The young Hawaiians in this study were born and raised in the blended Native and Western society of Hawai'i today. The students linked "being active with daily living" to knowledge of Hawaiian society before Western contact, as identified in the comment: "Like in the olden days they picked their food. They played all the sports. No TV, nothing, 'cause everything was off the land and ocean and they were active and stuff."

Participants from three of the four focus groups indicated that having a healthy self-identity and spirit was essential to ideal health. They talked about being "prepared mentally" for a healthy life, "having a healthy conscience," and "being spiritually healthy." One student noted that physical fitness "uplifts your spirit."

Participants from three of the four focus groups discussed the importance of a healthy diet. Comparing diet with physical activity, one student said, ". . . both [are] equal, but diet is more important, because, without a healthy lifestyle and eating, you won't have strength [to] do your exercise and meet your responsibilities." Several students noted that the traditional Hawaiian diet of their ancestors — based on fish, taro, and vegetables — was very healthy. A single comment from one of four focus group participants reflected the current saturation of media markets with messages to look good and to quit smoking.

#### Theme 2: Supports for and barriers to healthy living

Many supports for and barriers to healthy living were identified. In terms of support, 18 of 32 students noted easy access to no-cost activities with comments such as, "Just go to the beach. It's free," "You can run, surf, swim, or paddle; you don't have to pay," and "I just want to dance hula and keep on dancing hula." Hawai'i enjoys a year-round pleasant climate and ample access to mountains and beaches. However, according to the students, the biggest barrier to increasing their physical activity levels was lack of time. Included in this category was lack of time to travel from school, to work, and to a place (beach, mountain, field, or facility) where they could engage in activity.

A second support, mentioned by 13 students, was "getting some routine" that included time for physical activity. These activities could include activities that are part of one's job or family responsibility, as well as making time for physical activity between classes or after work. However, students expressed difficulty in establishing a healthy routine during the transition years between high school and adulthood, with multiple responsibilities of work, school, and starting a family. Although students knew how to fit physical activities into the high school day, they were not sure how to fit these activities into their now much-busier days, as illustrated by this quote: "I used to use the gym at high school. I think that was better." Twelve students talked about the demands of juggling work, school, and home responsibilities, leaving no time for exercise. As one student said, "If you're going to stay outside and paddle canoe or hula or dive, that's not going to pay the rent or bills."

Students commented on modern barriers to being active through daily living and barriers to having a healthy self-identity. All students nodded in agreement when one student said:

A lot of our Native Hawaiian younger generation . . . lose[s] touch with our identity. Perhaps over the years we [have] become more Americanized or sucked into the mainstream culture . . . now there's like Gameboy and TV and radio and stuff like that. And now days, we don't raise all our food. We go to the store and buy it. And the food, too, like it [was] all healthy and stuff and now we've got fast food.

Twelve participants made statements such as, "I think I could do a lot more, but it's just sometimes I'm lazy," "I find myself making excuses so I don't have to exercise," and "I'm not motivated to make healthy choices, even when healthy choices are within easy reach." Only nine students talked about personal accountability, and 12 expressed a sense of invincibility. They commented that because health problems were not imminent, special efforts at diet and exercise were not important, as expressed by the comment, "Because we're so young, we think, 'Whatever. We're young. We're healthy. We can do whatever we want.'"

Conversely, nine students talked about the galvanizing power of a critical incident like having a personal illness or experiencing the illness of a family member. One student related that, "My mother has diabetes, she was diagnosed last year. So [my father] started to work out, and we all eat healthier." Half of the students talked about the importance of family and social support in lifestyle choices. Support included having a friend or social group to exercise with and being in an environment where knowledge about diet and exercise is shared.

#### Theme 3: Ideas for program planning

The focus groups provided valuable information about programs and services that would be attractive to Native Hawaiian young adults enrolled in community colleges. For example, 20 students expressed interest in a program that incorporated information on Native Hawaiian heritage. All of the students nodded in agreement with notable excitement about culturally based opportunities. One student stated

If you put a cultural influence into it, it will make more Hawaiians our age push toward trying to interact with the program and want to be, you know [healthy] . . . If there was something like that I would, I mean, I would make time to do it.

Another student said, "I think if you do some kind of active program based in Hawaiian ways, it grounds you back home. It makes you . . . spiritually healthier." Another suggested, "I think a good program would have to incorporate Hawaiian heritage with working out."

When asked how such a program should be offered, 19 students suggested offering a credit-bearing course, tuition waivers, or other educational incentives. However, 16 of these students remarked that a course should be open to families and should encourage the building of family and social networks to increase attendance and, thus, increase the likelihood of sustained behavior change. As one student stated, "I think getting parents involved, I think that would help a lot . . . I think groups . . . it's more as a group than as individuals."

Fourteen students recommended a course that provides active learning strategies such as engaging students in growing and cooking healthy foods, reading nutrition labels, exercising as a group, and planning a personalized exercise program with a physical trainer. Nine students suggested didactic sessions to increase their knowledge about energy balance and health as well as to provide opportunities for reflection.

We asked specifically about the five CDC core prevention strategies to increase physical activity: 1) implementing community-wide campaigns, 2) conducting individual behavior change programs, 3) creating or enhancing access to places for physical activity, 4) implementing social supports in community settings, and 5) using point-of-decision prompts. Related to community-wide campaigns, students in two focus groups agreed that health programs should be advertised through newspaper, television, and radio. However, they noted that more Hawaiians would respond to celebrity endorsements, personal invitations, and word-of-mouth information. Regarding individual behavior change programs, students recommended group-oriented programs more often than individually oriented programs. Social support in community settings and creating and enhancing access to places for physical activity were overwhelmingly recommended as powerful strategies for change. Students laughed at point-of-decision prompts to use the stairs, for example, and reminded the moderator that LCC has only one set of stairs, located outside where a sign would be easily destroyed by blustery winds and rain.

### Key Informants

#### Theme 1: Ideal health

The composition of the traditional diet was approximately 10% protein, 10% fat, and 80% carbohydrate (39), and key informants noted that a traditional Hawaiian life of subsistence farming, fishing, and hunting was very active. Although they agreed with students that walking, playing with children, and having a physical job could be considered as physical activity, they doubted that students were really getting enough exercise through these means. They were intrigued by the students' broader definition of health to include identity and spirit. Thus, they recommend that programs for students include information about food quality (e.g., energy density) and exercise (e.g., duration, intensity, type) associated with both traditional and modern Hawaiian lifestyles. Informants suggested encouraging pursuit of traditional and free sports and engaging students to explore issues of identity and spirit in relationship to well-being.

#### Theme 2: Supports for and barriers to healthy living

Key informants were not surprised about student perceptions of invincibility (usual at this developmental stage), laziness, and difficulty incorporating physical activity into their routine, especially in Hawai'i, where the cost of living often necessitates that young people hold two or three jobs to make ends meet. However, they were encouraged to learn about the motivational potential of family- and peer-support systems. Although critical health incidences (i.e., personal illness or the illness or death of a close relative or friend) were motivating, key informants noted that these cannot be planned or promoted. Thus, programs need to encourage promotion of *ola pono* (i.e., healthy living) and *ola hāloa* (i.e., sustaining of life) within families and social groups (32). Key informants suggested that having students prepare a family health history could help them identify illnesses that run in their families, raising awareness of potential critical incidents.

#### Theme 3: Ideas for program planning

Key informants appreciated the students' request to incorporate Native Hawaiian cultural values and activities. They reminded us that Native Hawaiians are not the only people interested in preserving the Hawaiian culture; non-Native Hawaiians living in Hawai'i are also interested. Informants noted that incorporating cultural activities could attract more residents to healthy-living programs. They also agreed that a credit-bearing course made sense for this target group. They noted that the use of active-learning strategies was in line with native ways of learning, as Native Hawaiians traditionally learned by watching, doing, and teaching (21,36,37). Key informants appreciated the request to allow family members to participate and were willing to help brainstorm ways for this to occur within a community college system.

## Discussion

As recommended by the literature, talking to targeted end-users of healthy living interventions is important because recommending costly diets and structured exercise programs without first exploring a population's preferences is counterproductive (27-29,32,33). Although the applicability of this study to all Native Hawaiian college students is limited because of convenience sampling, this study helped us gain insight into how Native Hawaiian young adults enrolled in community colleges describe health and perceived supports for and barriers to healthy living.

Most participants described health and physical fitness as natural processes attributable to purposeful activities of daily living, while only a few participants equated physical fitness with intentional fitness activities (e.g., sports). For the most part, these Hawaiian students echoed the sentiment of their ancestors that good health comes from the purposeful activities of domestic life (36,37). In general, they did not believe that individually based strategies to promote physical activity would work, calling instead for group-based approaches that could fit into their busy days.

Participant-identified obstacles to healthy living were consistent with obstacles identified in current health promotion literature, including a decrease in physical activity during the transition from youth to adulthood and adulthood's associated multiple demands of job, college, home, and family (10). These barriers are common for students enrolled in community colleges because of their station in life (10,19). Students viewed a college-based course on fitness as a way to overcome some of these barriers. LCC CAG members were willing to explore opportunities to infuse culturally supported health messages into the university's required system-wide Hawaiian Studies 107 course, which is intended to promote cultural awareness among all students. Faculty members were willing to explore adding a healthy lifestyle course, combining nutrition education and energy balance, as a science elective.

Recent innovations in health promotion have included infusion of health issues into curricula. For example, Riley et al found that students exposed to health risk behavior information infused into the curriculum of an undergraduate course reported an increased awareness about health education resources and modified individual risk behaviors (40). Other researchers found that requiring an introductory health and physical education course increased the health knowledge, attitudes, and behaviors of college students (41) and alumni later in life (42).

Given the erosion of Native Hawaiian culture and students' belief in the importance of cultural identity and spiritual well-being to health, students wanted the course to have a contextual basis of Hawaiian traditions and activities, including farming, paddling, and hula. Benefits to enhancing participation by incorporating Hawaiian values and traditions have been found by other researchers (5,21-23,37). This request by students was in line with recommendations by key informants to combine diet and exercise counseling with behavioral strategies based on traditional learning styles in culturally relevant settings. Key informants affirmed the importance of imparting knowledge and skills about healthy living to help young Hawaiians as they begin to relabel themselves as a wellness-conscious people.

Economic incentives in the form of tuition waivers and college credits are attractive to this group. The literature shows that resources to support the health and fitness needs of college students who face the greatest barriers are scarce (19). Efforts to improve the health status of members of ethnic and racial minority groups, including Native Hawaiians, should be combined with efforts to improve their educational levels as part of a broad approach to decreasing known risks for chronic disease and morbidity. Information about this type of approach was not found in the literature; however, economic incentives in primary care are effective in the short run for simple, well-defined behavioral goals, and even small incentives can produce finite changes (43). The possibility of creating a tuition-assisted or tuition-waived health promotion and physical activity course for credit, funded with students' health fees, should be explored.

## Figures and Tables

**Table 1:** Demographic Characteristics of Native Hawaiian Focus Group Participants (N = 32), O'ahu, Hawai'i, 2005

Characteristic	No. of Participants (%)
**Age, y**	
18-20	17 (53.1)
21-25	15 (46.9)
**Sex**	
Female	20 (62.5)
Male	12 (37.5)
**Marital status**	
Single	30 (93.8)
Married or cohabiting	2 (6.2)
**No. in household**	
1	2 (6.2)
2	4 (12.5)
3-4	11 (34.4)
5-7	9 (28.1)
≥8	6 (18.8)
**Employment status**	
Full time	5 (15.6)
Part time	14 (43.8)
Unemployed	13 (40.6)
**Educational status**	
Just completed HS and enrolling in college	13 (40.6)
Fewer than 30 college credits (completed <2 years of college)	9 (28.1)
More than 30 college credits (completed >2 years of college)	10 (31.2)
**College major**	
Health sciences	8 (25.0)
Education	2 (6.2)
Liberal studies	7 (21.9)
Business/computer	4 (12.5)
Vocational	5 (15.6)
Undecided	6 (18.8)

**Table 2:** Self-Rated Health and Exercise Behaviors of Native Hawaiian Focus Group Participants (N = 32), O'ahu, Hawai'i, 2005

Characteristic	No. of Participants (%)
**Self-rated health**	
Very unhealthy	0
Somewhat unhealthy	3 (9.4)
Unsure	6 (18.8)
Somewhat healthy	21 (65.6)
Very healthy	2 (6.2)
**No. of days per week exercise is performed**	
0	4 (12.5)
1-2	4 (12.5)
3-4	10 (31.2)
5-7	14 (43.8
**No. of minutes spent exercising per day**	
0	4 (12.5)
1-29	10 (31.2)
30-59	9 (28.1)
≥60	9 (28.1)
**Meets physical activity recommendation^a^**	
Yes	13 (40.6)
No	19 (59.4)

a Based on Centers for Disease Control and Prevention's recommendation of at least 30 minutes of moderate-intensity physical activity on 5 or more days per week.

**Table 3:** Themes and Subthemes Generated During Focus Groups, by Number of Participants and Number of Focus Groups, O'ahu, Hawai'i, 2005

Themes and Subthemes	No. of Participants (%) (N = 32)	No. of Focus Groups (N = 4)
**Ideal health**		
Being active through purposeful living	20 (62.5)	4
Having a good self-identity	9 (28.1)	3
Following a healthy diet	7 (21.9)	3
**Supports for healthy living**		
Free activities	18 (56.2)	4
Routine that includes exercise or hard work	13 (40.6)	3
Social supports	18 (56.2)	4
**Barriers to healthy living**		
Lack of time, travel time	15 (46.9)	4
Living in today's society	12 (37.5)	4
Laziness, sense of invincibility	12 (37.5)	4
**Ideas for program planning**		
Offer course in Native Hawaiian context	20 (62.5)	4
Incentives, credits, tuition waivers	19 (59.4)	4
Social support	16 (50.0)	3
Active learning	14 (43.8)	4
